# Atrial fibrillation classification based on the 2D representation of minimal subset ECG and a non-deep neural network

**DOI:** 10.3389/fphys.2023.1070621

**Published:** 2023-02-14

**Authors:** Hua Zhang, Chengyu Liu, Fangfang Tang, Mingyan Li, Dongxia Zhang, Ling Xia, Stuart Crozier, Hongping Gan, Nan Zhao, Wenlong Xu, Feng Liu

**Affiliations:** ^1^ School of Information Technology and Electrical Engineering, University of Queensland, Brisbane, QLD, Australia; ^2^ School of Instrument Science and Engineering, Southeast University, Nanjing, Jiangsu, China; ^3^ Zhejiang Provincial Centre for Disease Control and Prevention CN, Hangzhou, Zhejiang, China; ^4^ Department of Biomedical Engineering, Zhejiang University, Hangzhou, Zhejiang, China; ^5^ School of Software, Northwestern Polytechnical University, Xi’an, China; ^6^ Department of Biomedical Engineering, China Jiliang University, Hangzhou, Zhejiang, China

**Keywords:** atrial fibrillation identification, electrocardiogram, recurrence plot, non-deep neural network, optimal subset

## Abstract

Atrial fibrillation (AF) is the most common cardiac arrhythmia, and its early detection is critical for preventing complications and optimizing treatment. In this study, a novel AF prediction method is proposed, which is based on investigating a subset of the 12-lead ECG data using a recurrent plot and ParNet-adv model. The minimal subset of ECG leads (II &V1) is determined *via* a forward stepwise selection procedure, and the selected 1D ECG data is transformed into 2D recurrence plot (RP) images as an input to train a shallow ParNet-adv Network for AF prediction. In this study, the proposed method achieved F1 score of 0.9763, Precision of 0.9654, Recall of 0.9875, Specificity of 0.9646, and Accuracy of 0.9760, which significantly outperformed solutions based on single leads and complete 12 leads. When studying several ECG datasets, including the CPSC and Georgia ECG databases of the PhysioNet/Computing in Cardiology Challenge 2020, the new method achieved F1 score of 0.9693 and 0.8660, respectively. The results suggested a good generalization of the proposed method. Compared with several state-of-art frameworks, the proposed model with a shallow network of only 12 depths and asymmetric convolutions achieved the highest average F1 score. Extensive experimental studies proved that the proposed method has a high potential for AF prediction in clinical and particularly wearable applications.

## 1 Introduction

Atrial fibrillation (AF) is a supraventricular tachyarrhythmia caused by uncoordinated atrial electrical activation and ineffective atrial contraction (Hindricks et al., 2021). As the most common cardiac arrhythmia and a major risk factor that can lead to ischemic, the AF incidence and prevalence have increased over the last 20 years, becoming one of the largest epidemics and public health challenges (Lippi et al., 2021). The diagnosis of AF at an early stage is essential for the timely inception of treatment, which is usually realized by analyzing Electrocardiogram (ECG) signals. In clinical practice, the body surface ECG is a powerful tool to reveal the occurrence, maintenance, and termination of AF. However, manual analysis of continuous rhythm registrations is time-consuming and needs cardiologists with expertise in ECG-based diagnosis.

In recent years, automated AF detection based on traditional methods and neural networks has been actively developed (Wesselius et al., 2021). Traditional methods mainly focus on atrial and ventricular signal features obtained from single-lead or standard 12-lead ECG recordings. The atrial features are primarily based on the P-wave disappearance or f-waves appearance. Typical methods include the wavelet energy method (Garcia et al., 2016; Serhal et al., 2022), the frequency and amplitude features of the f-wave (Henriksson et al., 2018), and the time between *P*-waves as a measure of the atrial rate (Huang et al., 2020). The ventricular features mainly describe irregularity of intervals between subsequent R-peaks (R-R intervals). Conventional methods also study wavelet sample entropy (Serhal et al., 2022), normalized fuzzy entropy (Liu C. et al., 2018), Shannon entropy (Dharmaprani et al., 2018), R-R interval features (Lown et al., 2020; Luo et al., 2021), and heart rate variability analysis (Nguyen et al., 2018). The signal features describe other characteristics buried in ECG and are related to AF’s clinical presentation and pathophysiology (e.g., signal quality and frequency components). A bimodal analysis of physiological time and frequency components is used to detect AF (Kruger et al., 2019). The ECG signals are transformed into the frequency domain (Khadra et al., 2005), time-frequency domain (Asgari et al., 2015), and phase space (Parvaneh et al., 2018) to predict AF.

The standard 12-lead ECG provides a complete evaluation of cardiac electrical activity, commonly employed across clinical settings. Existing neural network studies have mostly addressed the task of automatic AF classification based on the standard 12-lead ECG in different ways. For example, Ribeiro et al. presented a DNN framework to diagnose AF and other five types of rhythms recordings with an F1 score above 80% (Ribeiro et al., 2020). Yao et al. developed an attention-based time-incremental convolutional neural network to detect AF and other arrhythmias from the 12-lead ECG with varied-length (Yao et al., 2020). Zheng et al. proposed an optimal multi-stage arrhythmia classification approach to predict AF and other types at a cardiologist-level accuracy (Zheng et al., 2020). Many works developed neural network methods based on the popular dataset from the first China Physiological Signal Challenge 2018 involving AF and other eight types of different rhythms (Runnan He et al., 2019; Tsai-Min Chen et al., 2020).

However, information redundancy exists in standard 12-lead ECG signals, which could induce systematic overfitting in deep learning, causing poor generalization, performance, and unnecessary computational costs. Thus, some recent studies have explored the optimal selection of ECG leads for cardiac arrhythmia classification. Lai et al. proposed a deep learning model using the optimal 4-lead subset that outperformed the classification performance of the complete 12-lead ECG on normal and eight arrhythmias (Lai et al., 2021). References (Jimenez-Serrano et al., 2022; Xu et al., 2022) used deep learning-based methods to discriminate multiple cardiac conditions with various lead combinations, namely six leads (I, II, III, aVR, aVL, aVF), four leads (I, II, III, V2), three leads (I, II, V2) and two leads (I, II) vs the standard 12-lead ECG, and the data were provided during the PhysioNet/Computing in Cardiology Challenge 2021. In our previous work (Zhang et al., 2021), we addressed the classification of AF and eight other types of arrhythmias utilizing RP representation of ECG signals based on the identified optimal leads (lead II and aVR) *via* the Inception-ResNet V2 framework in which general optimal leads were selected for nine types of arrhythmia classification. These earlier works explored the optimal ECG-lead subsets on multiple prevalent arrhythmias classification tasks.

AF prediction has recently been investigated based on single-lead ECG data. Hannun et al. developed a deep neural network to classify 12 rhythm classes, including AF and other arrhythmias, based on single-lead ECG records obtained from an ambulatory monitor with high diagnostic performance, similar to cardiologists (Hannun et al., 2019). Ma et al. proposed a multi-step method that combined the support vector machine classifier and an auto-encoding network to predict the paroxysmal AF based on single-lead long-term ECG data from the fourth China Physiological Signal Challenge (CPSC 2021) database (lead II) and the wearable ECG database collected by the wearable ECG device (Ma et al., 2022). Athif et al. proposed an algorithm to discriminate AF from normal and other arrhythmias based on a short single-lead ECG (lead I), obtained from the Computing in Cardiology Challenge 2017 (Clifford et al., 2017). Mathunjwa et al. developed an approach to classify AF from VF, PAC, and PVC arrhythmia in two steps using a convolutional neural network based on the datasets from the MITDB, MIT-NIH AFDB, and MIT-BIH VFDB, in which the data is from the lead II recording channel (Mathunjwa et al., 2021).

Nevertheless, accurate diagnosis of AF using single-lead ECG data (lead I or II) is still challenging. Despite the above studies reporting promising AF detection results, one main challenge of these methods is the loss of certain morphologic features and patterns only visible in specific leads. For example, the low amplitudes of the f wave are mainly observable in lead V1 and aVF, whereas they barely appear in lead I (Cheng et al., 2013).

The clinical diagnosis of cardiac arrhythmia types is often task-specific. To improve AF detection performance and efficiency, it is essential to identify a minimal number of leads and which leads should be included in the analysis. In this work, we developed a novel method to explore the minimal subset of ECG leads dedicated to AF prediction. Furthermore, to achieve better classification results, we use the recurrence plot (RP) technique to represent ECG signals. The RP technique (Eckmann et al., 1987; Eckmann et al., 1995) has been widely used to explore the recurrence features and irregular cyclicities properties of time series dynamic information in the phase space. It is a visualization method that transforms the 1D time signals into 2D RP images (Izci et al., 2019). Zeng et al. developed a recurrence plot-based densely connected convolutional network to classify the epileptiform based on EEG (Zeng et al., 2021). Afonso et al. proposed an RP-based approach for identifying Parkinson’s disease (Afonso et al., 2019). The RP method was also combined with deep learning models for arrhythmias classification based on ECG (Zbilut et al., 2002; Mathunjwa et al., 2021; Zhang et al., 2021; Labib and Nahid, 2022).

Moreover, in this work, we attempted to achieve higher AF prediction performance with “non-deep” neural networks. In our previous study, we found that the Inception-ResNet V2 could enhance the diversity of the filter patterns by asymmetric convolution splitting, thus improving arrhythmia classification performance (Zhang et al., 2021). However, it requires training deep networks involving large-scale sequential processing and higher computing cost. This is challenging and less suitable for those applications requiring fast responses. Here, we improved the non-deep ParNet (Goyal et al., 2021) (Parallel Networks), combining the asymmetric filters for this RP-based AF prediction task.

The main contribution of our work is as follows: 1) A novel neural network method combining the recurrence plot technique and ParNet-adv model was proposed for AF classification. 2) We find the minimal subset of ECG leads for AF prediction. 3) We proposed a shallow network with only 12 depths and asymmetric convolutions for AF prediction. Our method, combined with a tailored ECG subset and a light framework, can be used as a screening tool for automatic and early detection of AF problems, particularly useful for portable or wearable ECG devices.

The rest of the paper is organized as follows: methods and materials are described in Section 2, experiments and results are detailed in Section 3 and 4, validation of the proposed method is provided in Section 5, a discussion is presented in Section 6, and conclusions are drawn in Section 7.

## 2 Methodology and materials

**FIGURE 1:**
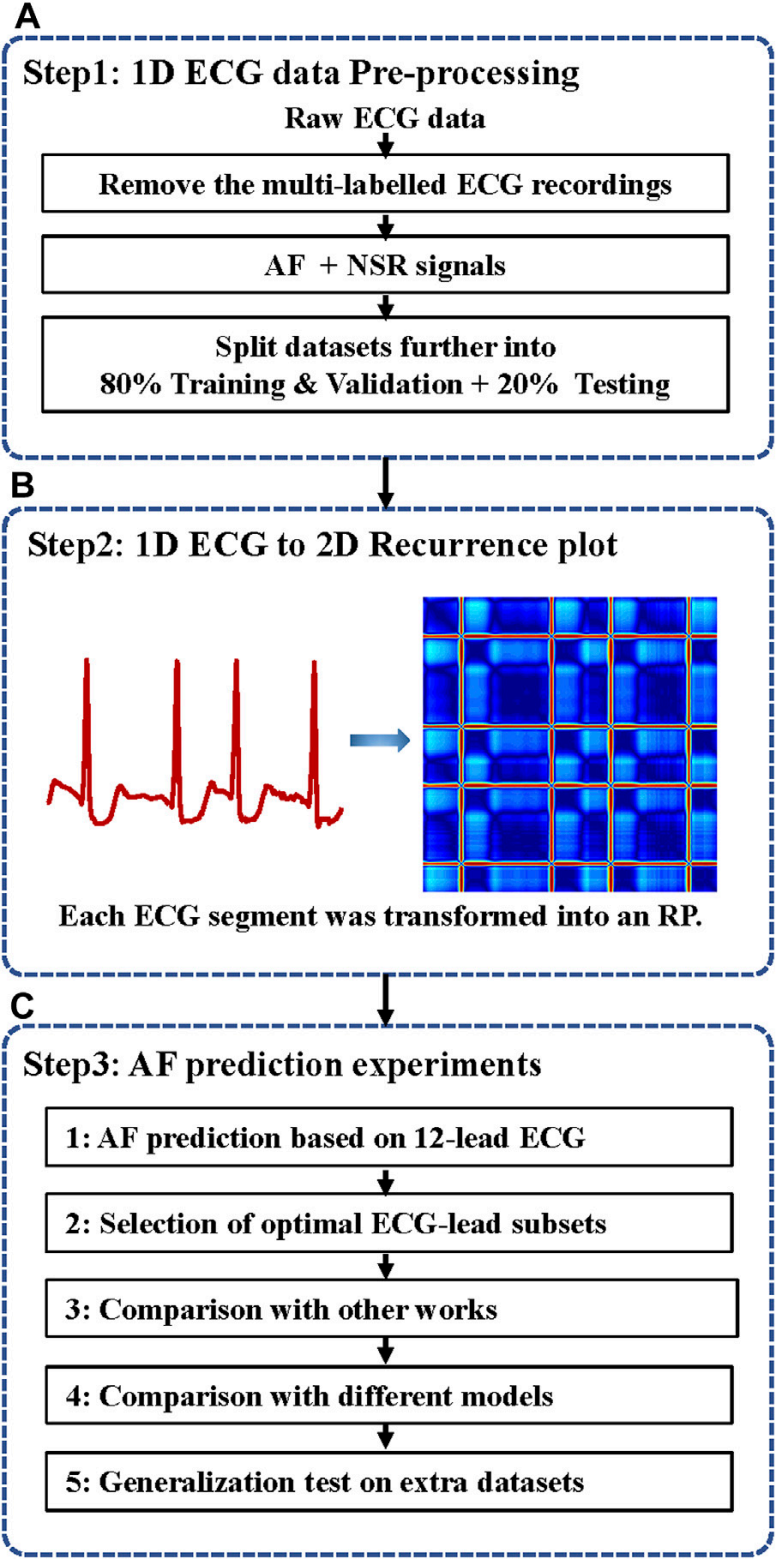
The flow chart of the automatic AF prediction system. ECG, electrocardiogram; AF, atrial fibrillation; NSR, normal sinus rhythm; 1D, one dimensional; 2D, two dimensional; RP, recurrence plot. **(A)** ECG data pre-processing. **(B)** ECG data were transformed into RP images. **(C)** AF prediction experiments based on 12-lead ECG and selected the minimal ECG-lead subsets. The validation superiority of the proposed method and testing the generalization on different extra databases.

In this work, we develop a novel neural network method for ECG-based AF prediction. The method selects the minimal subset ECG leads for AF prediction by combining the light ParNet-adv architecture and the recurrence features buried in AF and normal ECG signals. As shown in Figure 1, the system includes three steps: 1D ECG data pre-processing, conversion of 1D ECG into 2D RP images, and AF prediction.

### 2.1 ECG database

The dataset Physikalisch-Technische Bundesanstalt (PTB-XL) (Wagner et al., 2020) was used for training, validation, and testing. Another two ECG datasets (including CPSC and Georgia) were used to evaluate the generalization of the proposed approach. The data source CPSC (Liu F. et al., 2018) is the public training dataset from the China Physiological Signal Challenge (CPSC 2018). Georgia is a 12-lead ECG Challenge Database, Emory University, Atlanta, Georgia, United States, representing a large population from the South-eastern United States. These datasets were publicly accessible from the PhysioNet/Computing in Cardiology Challenge 2020 (Perez Alday et al., 2021) and detailed in Table 1. Each data contains 12-lead ECG recordings (I, II, III, aVL, aVR, aVF, V1–V6) sampled at 500 Hz with the mean duration of 10 s for PTB_XL and Georgia, and 16.2 s for CPSC.

**TABLE 1:** The profile of ECG Datasets.

Datasets	Sample frequency (Hz)	Mean duration (s)	ECG leads	CA types		Number of data	Single-label data	Experiment data
PTB-XL	500	10	12	NSR		18,092	16,801	1,200
				AF		1,514	1,396	1,200
CPSC	500	16.2	12	NSR		918	918	918
				AF		1,221	1,000	1,000
Georgia	500	10	12	NSR		1752	1,000	1,000
				AF		570	527	527

### 2.2 Data pre-processing for network input

#### 2.2.1 1D ECG data pre-processing

In the data pre-processing stage, as illustrated in step 1 of Figure 1A, the data with multi-labels were removed to focus on the single-labelled AF classification. 16,801 Normal sinus rhythm (NSR) and 1396 AF in the PTB-XL, 918 NSR and 1000 AF in the CPSC, and 1000 NSR and 527 AF in Georgia were obtained after data-pre-processing. The proportion of AF and NSR is unbalanced in PTB-XL. To balance the data proportion, 1200 AF and NSR data were randomly picked up. Four in five of the data labelled AF(NSR) were used as the training & validation dataset, and one in five was used as the test dataset. Thus, the training & validation set is independent of the testing set without overlap, usually called inter-patient classification (Huang et al., 2014). Afterward, each ECG data was split into 12 subsets corresponding with the 12 leads.

Converting 1D ECG signals to 2D Recurrence plot (RP) images.

Cardiac activity has temporal evolutions, including polarization and depolarization, which can be considered as a dynamic system (Labib and Nahid, 2022). Using electrodes, ECG records dynamic features of the cardiac electrical activities in the form of time-varying voltages, which is not easy to visualize whole aspects of the system dynamics in the time domain (Debayle et al., 2018). A recurrence plot (RP) is a widely used graphical tool to visualize the recurrent behaviors of the time series in phase space (Eckmann et al., 1995). It enables analyzing the dynamic recurrence features buried in ECG. The RP is obtained as follows.

Step 1: A 1D time series $X\left(t\right)$ phase space reconstruction is performed *via* Takens’ delay coordinate method (Takens, 1981). One consecutive time series is generated from the original time series, where τ is a constant delay taken as 1.

$$Y\left(t\right)=X\left(t-\tau \right)$$ (1)

Step 2: The 2D phase space trajectory is constructed from X and Y . The constructed vector is generated in the phase space as S_1_(x_1_, y_1_), S_2_(x_2_, y_2_), ……S_n−1_ (x_*n*−1_, y_*n*−1_) (Debayle et al., 2018).

Step 3: The distance between $S_{i}$ and $S_{j}$ on the trajectory can be formulated as:

$$R_{i,j}=\theta \left(\varepsilon -\left\|S_{i}-S_{j}\right\|\right),i,j=1,....,N$$ (2)

Where N is the length of the time series, ε is a threshold distance, $\left\|\cdot \right\|$ is a norm (e.g., Euclidean norm), and θ (.) is the Heaviside function and defined as:

$$\theta \left(Ζ\right)=\left\{\begin{array}{c} 0,if\;Z<0\;\\ \;1,otherwise \end{array}\right.$$ (3)

As a result, an RP image is obtained based on the matrix $R_{i,j}$ , which is a reconstructed recurrence representation in 2D phase space. As can be seen from Eq. 2, the RP is a binary matrix because of the threshold distance ε . This processing may lose some detailed information. In this work, an un-threshold approach proposed by (Faria et al., 2016) was adopted to avoid information loss by the R-matrix binarization, to obtain an RGB image, and to make use of the color information in RP images. Then the R-matrix can be defined as:

$$R_{i,j}=\left\|S_{i}-S_{j}\right\|,i,j=1,....,N$$ (4)

In the present study, as illustrated in Figure 1B, the 1D ECG signals were converted to 2D RP images as the input signals of the 2D network for AF prediction.

### 2.3 ParNet-adv-based AF classification

In this work, we modified the ParNet (Goyal et al., 2021) (Parallel Networks) as a “non-deep” neural network for this RP-based AF prediction task. The classification network with a shallow depth and asymmetric filters is called ParNet-adv. The schematic architecture of the ParNet-adv used for AF prediction is represented as follows.

**FIGURE 2:**
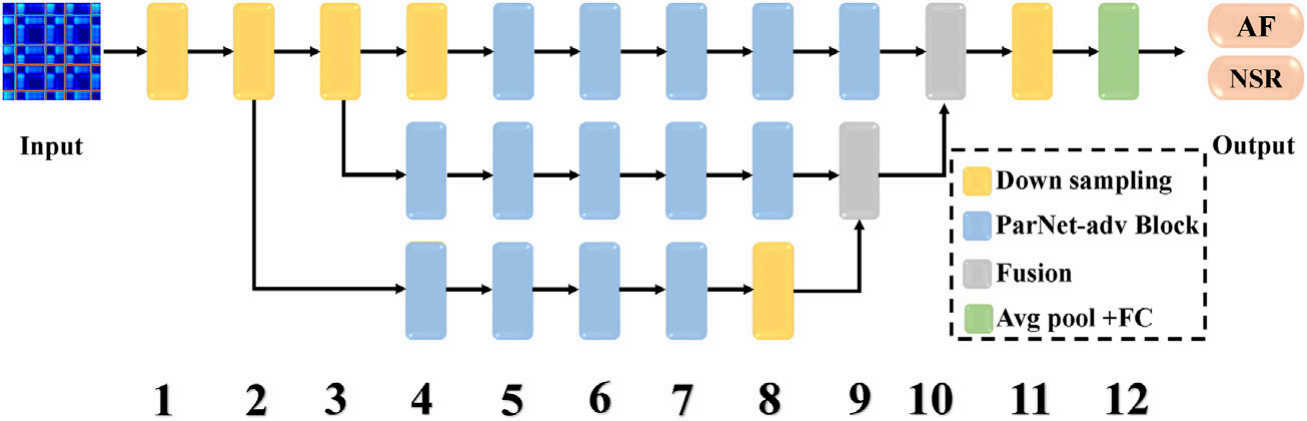
The architecture of the ParNet-adv for AF prediction. It consists of three parallel streams and four sub-networks, including ParNet-adv Block, Downsampling, Fusion, Avg pool + FC. The ParNet-adv model has only 12 depths of layers, the model inputs are RP images, and the outputs are the predictions of AF and NSR. AF, atrial fibrillation; NSR, normal sinus rhythm.

**FIGURE 3:**
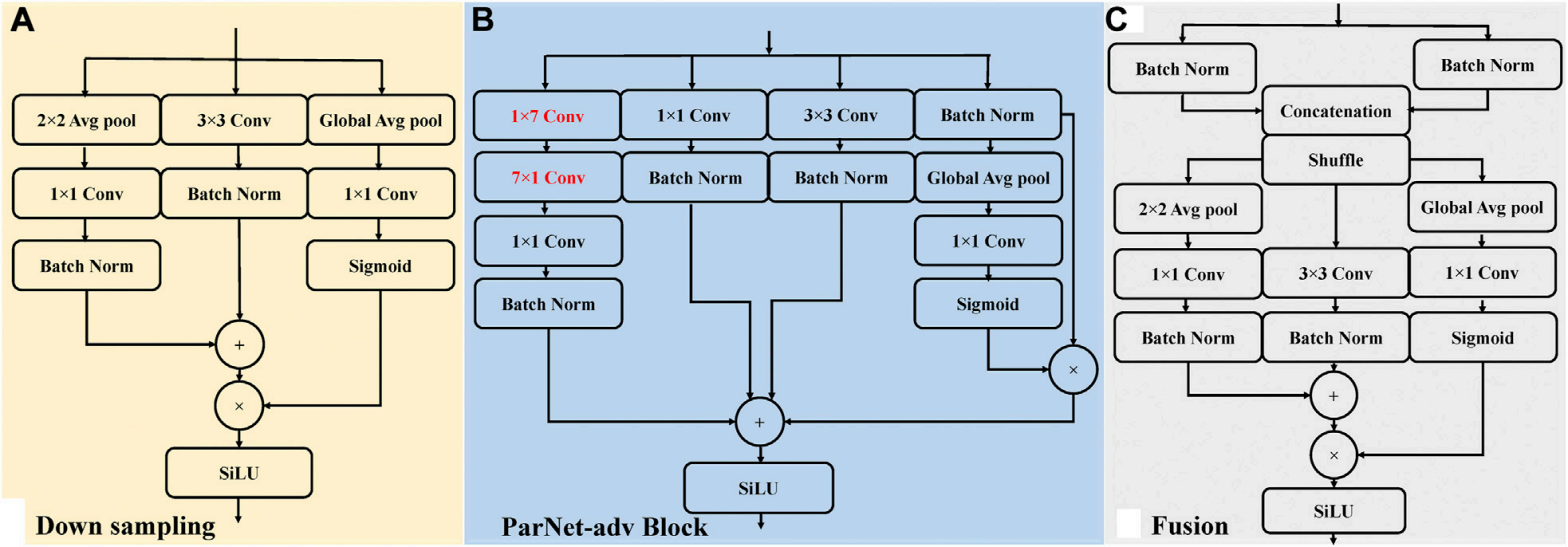
The sub-networks architecture of the ParNet-adv model, including ParNet-adv Block, Downsampling, and Fusion. Conv, convolutional layer; Batch Norm, batch normalization layers; Global Avg pool, global average pooling layer; Avg pool, intermediate pooling layer; SiLU, sigmoid linear unit activation. **(A)** An illustration of the Downsampling block. **(B)** An illustration of the ParNet-adv block with the key design of 1 × 7 and 7 × 1 asymmetric convolutions. **(C)** An illustration of the Fusion block.

As illustrated in Figure 2, the shallow ParNet-adv model with a depth of 12 layers is a parallel model with three streams, including four parallel sub-networks (Downsampling, ParNet-adv Block, Fusion, Avg pool + FC). Downsampling Blocks in Figure 3A reduce resolution and increase the width to enable multi-scale processing. For the ParNet-adv Block in Figure 3B, the key design choice is the use of 1 × 7 and 7 × 1 asymmetric convolutions. The ParNet has only 3 × 3 convolutions, which is challenging as the receptive field is somewhat limited. To address this, we build asymmetric filters inspiring from the Inception-ResNet V2 design with 1 × 7 and 7 × 1 convolutions providing a large and diverse reception scale in the proposed ParNet-adv model. Fusion Blocks in Figure 3C combine information from multiple resolutions. The Avg pool and FC Blocks perform AF classification. In addition, one concern is that a non-deep network may have insufficient non-linearity, limiting its representational power. Thus, the model replaces the ReLU activation with SiLU. In this work, we trained our networks with the cross-entropy loss, a learning rate of 0.001, a batch size of 64, and the RP input images with a resolution of 299 × 299.

### 2.4 Performance analysis of the proposed method

To assess the effectiveness of the proposed method, several parameters, including Precision, Recall, Specificity, Accuracy and F1 score are adopted, which are defined as follows.

$$Precision=\frac{TP}{TP+FP}$$ (5)

$$Recall=\frac{TP}{TP+FN}$$ (6)

$$Specificity=\frac{TN}{TN+FP}$$ (7)

$$Accuracy=\frac{TP+TN}{TP+TN+FP+FN}$$ (8)

$$F1=\frac{2\left(Precision\times Recall\right)}{Precision+Recall}$$ (9)

Where TP is the number of true positive data; FP is the number of false positive data; FN is the number of false negative data. Here, Precision is the fraction of all predicted data that are labelled data; Recall is the fraction of all labelled data that are successfully detected; Specificity is the probability of a negative test, conditioned on truly being negative; and Accuracy is the fraction of correct classifications. The F1 score among classes is computed to evaluate the model’s final performance.

## 3 Experiments

### 3.1 Experimental design and computing environment

As illustrated in Figure 1, we designed several experiments, including the selection of sampling frequency and length of ECG data, minimal leads selection, and comparison between the proposed method and conventional 12-leads and other leads options-based solutions. All experiments were conducted on Wiener nodes of the University of Queensland computer cluster with 4 * Nvidia Volta V100 SXM2 connected GPUs per node. Each node contains 5120 CUDA cores, 640 TensorFlow hardware cores, and 32 GB of HBM2 class memory. This model was implemented using the TensorFlow 3.6 and Karas deep learning framework. The fivefold cross-validation was introduced in the training and validation processing.

### 3.2 Selection of the sampling frequency and length of data

In this section, we compared the performance of AF classification based on different sampling frequencies and data lengths. Each original data was sampled at 500 Hz with a mean duration of 10 s. For comparison, we downsampled the lead II and lead VI of ECG data into 200 Hz, and 300 Hz. In addition, the data were split into 5 s and 10 s in length at each sampling frequency, respectively. Regarding the sampling frequency 200 Hz/300 Hz/500 Hz, we picked up 5 s segment of the data from first to 1000th/1500th/2500th, and 10 s segment from 1st to 2000th/3000th/5000th. Each ECG segment was transformed into the corresponding RP image, with the *z*-score normalization of the input signals of the model. The average F1 score was chosen for performance evaluation.

The results of these experiments are summarized in Table 2. The results suggest that almost all the performance of AF detection on 10 s data length are better than 5 s in three sampling frequencies, except the Recall of 500 Hz. Further, the experiment with the 300 Hz sample frequency and 10 s data length achieved the optimal F1 score and Accuracy over others. Based on this investigation, we downsampled the ECG signal to 300 Hz and picked up 10 s data for each recording to carry out the following AF detection experiments.

**TABLE 2:** Performance of AF classification based on different frequencies and data lengths.

Frequency (Hz)	Data length (s)	F1	Precision	Recall	Specificity	Accuracy
200	10	0.9738	0.9810	0.9667	0.9813	0.9740
	5	0.9625	0.9664	0.9583	0.9667	0.9625
300	10	0.9763	0.9654	0.9875	0.9646	0.9760
	5	0.9565	0.9506	0.9625	0.9500	0.9367
500	10	0.9718	0.9748	0.9688	0.9750	0.9719
	5	0.9598	0.9491	0.9708	0.9479	0.9594

### 3.3 Selection on minimal ECG-leads subset for AF detection

In this section, we determine which leads are necessary to keep and which carry redundant information that can be removed from the automated AF detection system. The ParNet-adv model was used to identify AF *via* analyzing recurrence features of RP images derived from the complete 12-leads ECG and minimal ECG-leads subset based on the PTB-XL dataset.

A forward, stepwise minimal subset selection method (James et al., 2013; Lai et al., 2021) was used to find an minimal ECG-lead subset for AF detection based on the same ParNet-adv model. In the first phase, we conduct an AF prediction based on each lead and find the one achieving the best performance. The selected lead will be set as the seed one in the minimal subset. In the second phase, the other 11 leads will individually combine the seed lead in phase one to undertake another round of AF prediction, from which we can identify the best two leads with the best performance. In the next phase, we repeat the search with the selected two leads from the first two phases. In each operation, we trained the model and tested the performance with the addition of each single-lead ECG into the minimal lead subset until finding that the incorporation of any single-lead ECG no longer improves the detection performance. We stop searching if we see further enhancement cannot be achieved. We use the fivefold cross-validation to train and test the classification performance each time. The matric F1 score was applied to measure AF prediction performance. And we conduct the two-sample *t*-test between every two groups’ F1 scores. Our null hypothesis is that the performance of the two groups is dependent. And our alternative hypothesis is that the performance of the two groups is independent. A *p*-value is used as a threshold to reject or accept the null hypothesis. In accordance with the acceptance of statistical significance at a *p*-value of 0.05 or 5%, CI is calculated at a confidence level of 95%. Among all steps, we choose the one that can achieve the optimal F1 score as the final minimal subset of 12-lead for AF prediction through the above multiphase searching procedure.

## 4 Results

**FIGURE 4:**
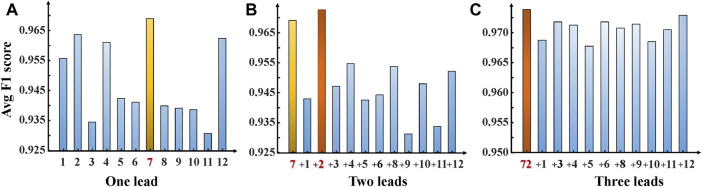
F1 scores—ECG leads bar chart. Show the performance F1 score for our ParNet-adv model on each AF prediction experiment. **(A)** One-lead AF prediction and show the lead V1 achieved the optimal performance. **(B)** Two-leads AF prediction (addition of each single lead to the lead V1) shows the highest F1 score bar corresponding to the lead V1+ II subset. **(C)** Three-leads AF prediction (addition of each single-lead to the subset leads V1+II). (1,2,3,4,5,6,7,8,9,10,11,12 stands for lead I, II, III, aVR, aVL, aVF, V1, V2, V3, V4, V5, V6).

**FIGURE 5:**
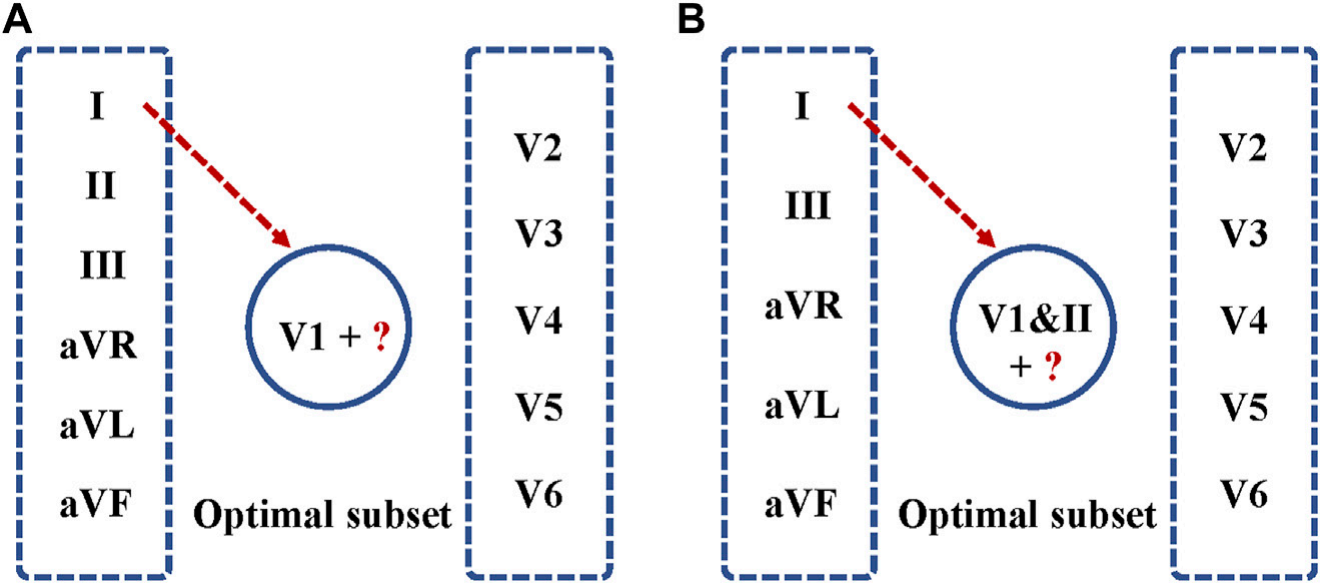
Optimal ECG lead subset selection for AF detection. **(A)** Lead V1 was taken as the base element, and other leads were considered as candidates. Each time, a single lead was added to the seed set for training, validation of the model, and testing. **(B)** Leads V1&II were selected as the base element, and the other 10 leads were considered as candidates for repeat searching.

This section presents experimental results for AF and NSR classification. Two different scenarios were designed for the study. First, the classification experiment based on the complete 12-leads ECG was performed, and achieved the F1 score of 0.9692, the precision of 0.9721, and the recall of 0.9663, the Specificity of 0.9722 and the accuracy of 0.9693 for AF detection based on the fivefold cross-validation experiments. Second, the minimal subset of ECG was explored for AF discrimination, including three phases. As illustrated in Figure 4A, in the first phase, the F1 score for AF detection using single leads ranged from 0.9308 (lead V5) to 0.9729 (lead V1), and the lead V1 obtained the best overall results compared to other leads, which was statistically significant (*p* < 0.05). In the second phase, Figure 5A shows that lead V1 was taken as the base element, and other leads were considered candidates. As illustrated in Figure 4B, the subset composed of lead II and lead V1 achieved the best overall results (F1 score 0.9763) for AF detection over other combinations. In the third phase, Figure 5B shows that we repeated searching with selected leads V1 and II, individually combining every single lead from the other 10 leads. Among each step, the *p*-value is less than 0.05 and statistically significant. Therefore, we reject the null hypothesis and accept the alternative hypothesis that the performances of each two groups are independent. As illustrated in Figure 4C, incorporating more lead could not improve AF detection performance in this phase, and the F1 score of each experiment decreased. Thus, leads V1 and II were identified as the minimal subset of 12 leads ECG for AF detection.

## 5 Validation of the proposed method

### 5.1 Comparison of performance between cases with the minimal subset, the 12-lead, and the single lead (lead I or lead II) ECG signals

The performance of the minimal subset (leads V1 and II) was compared with that of other options (complete 12-leads, lead I, and lead II). Table 3 reports the comparison in terms of Precision, recall, Specificity, Accuracy and F1 score. Note that the performance with the minimal subset (F1 score 0.9763) (*p*-value <0.05) outperformed the performance based on the complete 12-lead (F1 score 0.9692), single-lead I (F1 score 0.9593), single-lead II (F1 score 0.9669) and single-lead V1 (F1 score 0.9729). It is noted that lead I, which is used in the Apple Watch (Perez et al., 2019), Karadia Mobile (Goldenthal et al., 2019), and single time point testing (Duarte et al., 2020) for AF detection; and lead II, which is used as the input signal to predict AF in (Mathunjwa et al., 2021; Ma et al., 2022), achieved ordinary performance in our study.

**TABLE 3:** Performance of AF classification based on different ECG leads.

ECG leads	F1	Precision	Recall	Specificity	Accuracy
I	0.9593	0.93655	0.9833	0.9333	0.9583
II	0.9669	0.9590	0.9750	0.9583	0.9667
V1	0.9729	0.9749	0.9708	0.9750	0.9729
II + V1	0.9763	0.9654	0.9875	0.9646	0.9760
12 leads	0.9692	0.9721	0.9663	0.9722	0.9693

### 5.2 Comparison with state-of-art models

In this section, we compared the proposed method with several state-of-art models based on the minimal subset as the input. For a fair comparison, we have trained the Inception-ResNet V2 (Szegedy et al., 2017), ParNet (Goyal et al., 2021), and the proposed ParNet-adv model with the same set of hyperparameters and input data. As illustrated in Table 4, the proposed method achieved the F1 score of 0.9763, higher than other reference models. In the study, we built the 1 × 7 and 7 × 1 layers based on the asymmetric design, increasing the receptive field of the ParNet-adv model, thus improving the performance than that of the ParNet performance with the same depth. In addition, note that the proposed model effectively reduces depth while can perform competitively with the deep model, the Inception-ResNet V2, in AF detection study (Table 4).

**TABLE 4:** Comparison of AF detection based on different models.

Model	Depth	Kernel size	F1	Precision	Recall	Specificity	Accuracy
Inception-ResNet v2 (Szegedy et al., 2017)	164	1 × 7,7 × 1 1 × 3,3 × 1	0.9752	0.9672	0.9833	0.9667	0.9750
ParNet (Goyal et al., 2021)	12	3 × 3	0.9700	0.9630	0.9771	0.9625	0.9698
ParNet-adv	12	1 × 7,7 × 1	0.9763	0.9654	0.9875	0.9646	0.9760

### 5.3 Generalization of the proposed method

In this section, we evaluated the generalization of the proposed method *via* testing two different ECG datasets CPSC (Liu F. et al., 2018) and Georgia (Perez Alday et al., 2021). The detailed information of these datasets is illustrated in Table 1. For proper testing, all data were pre-processed and fed into the model training, validation, and testing in the same way. As shown in Table 5, the proposed method achieved the F1 score of 0.8660 on CPSC and 0.9693 on Georgia based on the minimal subset of ECG leads (leads II and V1). These testing results indicate that the new method has a good generalization ability for AF prediction.

**TABLE 5:** Performance of AF classification based on the CPSC and Georgia ECG datasets.

Dataset	F1	Precision	Recall	Specificity	Accuracy
CPSC	0.9693	0.9518	0.9875	0.9454	0.9674
Georgia	0.8660	0.8702	0.8619	0.9322	0.9079
PTB-XL	0.9763	0.9654	0.9875	0.9646	0.9760

## 6 Discussion

In this study, we developed a neural network-based system for automatic AF prediction. The design incorporates several novel points: 1) it identifies which leads of 12-lead ECG are necessary for detecting AF features; 2) it uses RP images to train the neural network instead of 1D ECG data for better capturing the recurrence features of AF; 3) the neural network employs a light ParNet-adv structure, suitable for applications demanding a prompt response.

The results show that using the minimal ECG-lead subset outperformed the complete 12-lead ECG, supporting our hypothesis that eliminating the data redundancy can reduce the overfitting issue and thus improve the prediction performance. Note that the clinical diagnostic criteria of cardiac arrhythmia types are often lead-specific. So, the proposed algorithm explicitly seeks the minimal ECG-lead subset for AF prediction, and the selection is performed based on the most common short-time 12-lead ECG in the clinical setting. As demonstrated in Section 4, a minimal subset ECG lead (leads II & V1) can interpret AF rhythm with a significant increase of F1 score compared with the complete 12-leads ECG and other options.

The minimal lead subset obtained by this data-driven approach provides valuable insights for recurrence features in this automatic AF detection protocol. As a 2-lead subset, it consists of the limb lead II and the other unipolar lead V1, providing assessments in the horizontal plane from the vantage points of the septal surface. These two quasi-orthogonal leads (leads II & V1) play a vital role in AF prediction. This is consistent with clinical practice: Lead II, favored among the 12 leads by physicians for a quick exam of an ECG recording due to its clearest signal, has decent overall performance in predicting AF. Lead V1 is used in the clinic to detect fibrillatory waves, which can be either fine or coarse. Of the 12 ECG leads, the lead V1 electrode is considered closest to the right atrium. It was obvious that lead V1 electrode position is right in front of the right atrial free wall and that the right atrium almost entirely conceals the left atrium from a V1 point of view. The f-waves in all patients were most dominant in this lead (Holm et al., 1998; Hsu et al., 2008).

**FIGURE 6:**
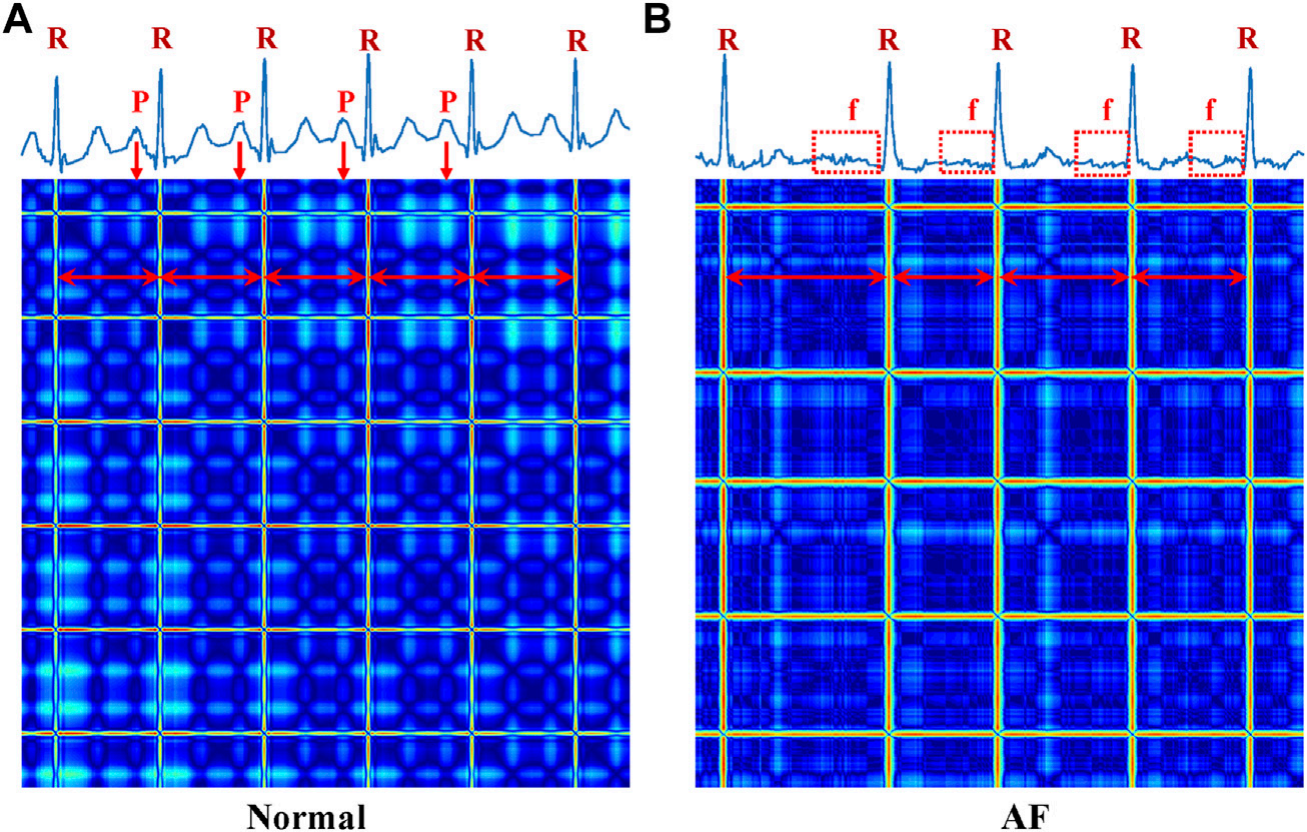
ECG time-series (up) and corresponding RP (below) images of Normal and AF. **(A)** Normal **(B)** AF. R, the R peak of the ECG; P, the P peak of the ECG; f, the f wave of the ECG.

Figure 6 shows the ECG time series and corresponding RP images. (A) is a normal ECG, the temporal waveform contains normal P waves, regular rhythm, and R-R interval, and the RP pattern shows the regular image texture. (B) represents an AF case, having features of missing the P waves and irregular RR intervals. The RP features were considered good predictors of AF (Huang et al., 2020), as they reflect the non-linear and non-stationary nature of the ECG signals. It has shown high efficiency in arrhythmia classification from the ECG signals, as demonstrated in our previous work (Zhang et al., 2021). In this study, we only extract recurrence features of a subset of RPs for AF detection. Note that fibrillatory waves are observable and present either fine or coarse, corresponding to irregular and cluttered textures in the RP of lead V1, as shown in Figure 6B. This corresponds well to the variation of f-waves recorded in lead V1 (see Figure 6A).

Regarding the feature extraction model, we introduced a novel shallow ParNet-adv network that integrated a non-deep ParNet with large and asymmetric filters of Inception-ResNet, to automatically extract high-quality recurrence structure features of RP images based on ECG leads. Therefore, our ParNet-adv model, integrated complementary advantages of these two networks (Table 4), is efficient for feature extraction and has achieved promising performance in AF detection. Thus, the ParNet-adv-like models have the potential to create an incredibly light recognition system for wearable applications. We also note that the study of ECG datasets of the PhysioNet/Computing in Cardiology Challenge 2020 has well demonstrated the generalization ability of the proposed method.

## 7 Conclusion

We have developed a novel neural network-based system for automatic AF prediction in this paper. The proposed method offers three main advantages. First, unlike most previous work, mainly based on single-lead ECG or standard 12-lead ECG data, this work performs AF detection with a minimal subset of leads (lead II &V1), thus more efficient and easier to implement than existing methods. Second, the proposed method achieved promising prediction performance using non-deep neural networks with only 12 depths. Third, the 1D ECG signals were transformed into 2DRPs for extracting structural topographies in images, beyond processing original time series. This solution is demonstrated useful for extracting signal dynamical features and better detecting AF. The benefits of the proposed method have been validated with extensive experiments; we hope this new method can be further improved for AF detection in clinical and wearable applications.

## Data Availability

Publicly available datasets were analyzed in this study. This data can be found here: https://physionet.org/content/challenge-2020/1.0.2/.
